# A prospective cohort study on effects of gemigliptin on cardiovascular outcomes in patients with type 2 diabetes (OPTIMUS study)

**DOI:** 10.1038/s41598-020-75594-5

**Published:** 2020-11-04

**Authors:** Eun Heui Kim, Sang Soo Kim, Dong Jun Kim, Young Sik Choi, Chang Won Lee, Bon Jeong Ku, Kwang Soo Cha, Kee Ho Song, Dae Kyeong Kim, In Joo Kim

**Affiliations:** 1 Department of Internal Medicine, and Biomedical Research Institute, Pusan National University Hospital, Busan, Republic of Korea; 2 Department of Internal Medicine, Inje University Ilsan Paik Hospital, Goyang, Republic of Korea; 3 Department of Internal Medicine, Kosin University College of Medicine, Busan, Republic of Korea; 4 Department of Internal Medicine, Busan St. Mary’s Hospital, Busan, Republic of Korea; 5 Department of Internal Medicine, Chungnam National University College of Medicine, Daejeon, Republic of Korea; 6 Division of Endocrinology and Metabolism, Konkuk University Medical Center, Konkuk University School of Medicine, Seoul, Republic of Korea; 7 Department of Internal Medicine, Inje University Busan Paik Hospital, Busan, Republic of Korea; 8 Department of Internal Medicine, Pusan National University Hospital, 179, Gudeok-ro, Seo-gu, Busan, Republic of Korea

**Keywords:** Endocrinology, Medical research

## Abstract

This study was performed to evaluate the long-term cardiovascular safety of gemigliptin in patients with type 2 diabetes mellitus (T2DM). After screening, eligible patients with T2DM were enrolled, received gemigliptin, and were followed up for a median of 2.50 years. The primary outcome was a composite of confirmed cardiovascular death, nonfatal myocardial infarction, or nonfatal ischemic stroke (3-point major adverse cardiovascular event [MACE]). The key secondary outcomes were incidence of all-cause mortality and any other cardiovascular events. A total of 5179 patients were included in the study and 5113 were treated with gemigliptin. Overall, the primary outcome occurred in 26 patients within 12 months (estimated incidence by Cox proportional hazard model 0.49%, 95% CI 0.29–0.69%) and in 54 patients within 54 months (estimated incidence from Cox proportional hazard model 1.35%, 95% CI 0.92–1.77%). During the study period, the incidence rates of each component of the primary composite outcome were 0.04% (0.2 events per 1000 person-years) for cardiovascular death, 0.51% (2.2 events per 1000 person-years) for nonfatal myocardial infarction, and 0.61% (2.5 events per 1000 person-years) for nonfatal ischemic stroke. The incidence of all-cause mortality was 0.82% (3.2 events per 1000 person-years) and the incidences of other cardiovascular events were all less than 0.3%. In conclusion, T2DM patients who received gemigliptin exhibited a low incidence of the primary composite MACE and all-cause mortality. Therefore, the use of gemigliptin is expected to be safe without an increase in cardiovascular risk.

Trial registration: The study was registered at ClinicalTrials.gov (identifier: NCT02290301).

## Introduction

The prevalence of type 2 diabetes mellitus (T2DM) has reached epidemic proportions globally and it is associated with cardiometabolic multimorbidity and mortality^1,2^. The goal of treatment is to achieve and maintain glycemic control to reduce the risks of macrovascular and microvascular complications associated with T2DM^3^. Given the heterogeneity of patients and the complementary mechanism of disease, different classes of antidiabetic agents have been developed and used for the management of hyperglycemia in T2DM^4,5^.

Dipeptidyl peptidase 4 (DPP-4) biologically activate a variety of bioactive peptides, including glucagon-like peptide-1 (GLP-1) and gastric inhibitory polypeptide^6^. DPP-4 inhibitors exhibit glucose-dependent insulinotropic effects and improve hyperglycemia with a low risk of hypoglycemia and other side effects^7^. Therefore, DPP-4 inhibitors have significantly changed the therapeutic options for patients in real-world practice around the world^8,9^. Gemigliptin, which is a DPP-4 inhibitor, stimulates the activity of incretin hormones by selectively inhibiting the activity of DPP-4 and exhibits 27,000- and 23,000- times greater selectivity than that DPP-8 and DPP-9, respectively^10^. The clinical effect of gemigliptin was shown to be superior to that of a placebo and non-inferior to comparable therapeutic agents in active use^11–13^.

Due to the issues related to the use of thiazolidinedione in 2007, the United States Food and Drug Administration and other regulatory authorities now require the results of cardiovascular (CV) outcome studies that investigate the long-term use of new diabetes drugs to confirm that they do not increase unexpected CV risks. Large-scale CV outcome studies were performed on the safety of DPP-4 inhibitors, and the results confirmed that these drugs do not increase CV risks^14–19^.

Important information supporting the CV safety of gemigliptin in patients with T2DM has been collected for the approved indications. However, data are still lacking on the long-term CV safety of gemigliptin, for usual care in patients with T2DM. This study was performed to evaluate CV events in patients with T2DM treated with gemigliptin monotherapy or in combination with other drugs.

## Methods

### Patients

Patients aged 19 years or older who were diagnosed with T2DM and scheduled to receive gemigliptin (single agent or fixed-dose combination) were included in this study. Key exclusion criteria included patients with type 1 diabetes, severe or end-stage heart failure (HF) (New York Heart Association class III or IV), or history of acute coronary syndrome or stroke within 3 months prior to screening, or who had taken any DPP-4 inhibitors or GLP-1 receptor agonists within 3 months prior to screening. Patients with any contraindications for gemigliptin were also excluded.

### Study design

This multicenter, single-arm, prospective cohort study was conducted at 149 centers in the Republic of Korea from June 2013 to November 2017 to assess the long-term CV safety of gemigliptin in patients with T2DM. The study was conducted in compliance with the ethical guidelines of the Declaration of Helsinki and Good Epidemiological Practices and was approved by the institutional review board of Pusan National University Hospital and 54 other study sites according to the standards of a regulatory authority. Written informed consent was obtained from all participants and the study was registered at ClinicalTrials.gov (NCT02290301).

All eligible patients were enrolled on Day 1. They received gemigliptin (single agent or fixed-dose combination) alone or in combination with other antidiabetics, and the regimen and dosage were determined at the discretion of the investigator. The patients were then followed up according to routine medical practice. The addition and discontinuation of, and changes in antihyperglycemic agents, including gemigliptin, were allowed during the study at the discretion of the investigator, taking into consideration the patient’s condition. All of the patients were followed up until the end of the study whenever possible, regardless of whether they took gemigliptin or other diabetic medications.

Patients were recruited for about 30 months, and the planned study participation period for each patient was at least 24 months. Therefore, the last enrolled patient could be followed up for at least 24 months, and the first enrolled patient could be followed up for about 54 months. All data of interest were collected every 6 months from usual care data. Patient who did not visit the study site for more than 6 months, were contacted by telephone to collect CV safety information.

### Outcomes

The primary composite major adverse CV event (MACE) endpoint was time to the first confirmed CV death, nonfatal myocardial infarction or nonfatal ischemic stroke. Investigators at the study site adjudicated all components of the primary composite endpoint and reported them as adverse events. All reported adverse events were coded by Medical Dictionary for Regulatory Activities (MedDRA) version 20.0. CV death was defined as death due to adverse events coded as myocardial infarction (narrow standardized MedDRA queries [SMQ]), central nervous system hemorrhage and cerebrovascular conditions (narrow SMQ), or unstable angina (preferred term [PT]). Nonfatal myocardial infarction was defined as an adverse event coded as myocardial infarction (narrow SMQ), and nonfatal ischemic stroke was defined as the adverse event term was coded as central nervous system hemorrhage and cerebrovascular conditions (narrow SMQ).

Secondary endpoints were classified as adverse events related to CV events and other adverse events. CV related endpoints included time to occurrence of the component of the primary composite MACE, the incidence of the primary composite MACE, incidence of each component of the primary composite MACE, incidence of all-cause mortality, and incidence of any other CV events (HF, hospitalization due to revascularization, peripheral vascular disease and unstable angina pectoris). Other endpoints related to adverse events included the incidences of any malignancies and any other specific adverse events (pancreatitis, increased blood amylase, increased lipase, arthralgia, bacteriuria, hypersensitivity and severe skin reactions such as Stevens-Johnson syndrome). Changes in glycated hemoglobin (HbA1c) levels from the baseline were included in other endpoints.

### Statistical analysis

The sample size was calculated by using a one sample survival test. Assuming an annual incidence of 2% for the primary composite MACE in patients with T2DM and a dropout rate of 10% before the occurrence of MACE, the sample size of approximately 5000 patients was required to detect a 20% reduction of risk when treated with gemigliptin at a power of 95% and a significance level of 5%.

Statistical analyses were performed on the safety set, which included all participants who received gemigliptin at least once and who were followed up more than once.

Continuous and categorical data were summarized as descriptive statistics. The time to occurrence of the primary composite MACE was analyzed using a Cox proportional hazard model and Kaplan–Meier curves were plotted. The incidence of the primary composite MACE was calculated using a Cox regression model that included age, sex, duration of diabetes, and smoking status, which were expected to be related to MACE, as covariates. If the upper limit of the 95% CI of the annual incidence was less than 2%, gemigliptin was considered not to increase the incidence of the primary composite MACE. The incidence, 95% CI, and incidence rate (per 1000 person-years) were calculated for all endpoints except HbA1c level, and survival analysis was performed on the time to death. The paired *t*-test or Wilcoxon’s signed-rank test was performed to test for changes in HbA1c levels.

Additional subgroup analyses were performed based on the following treatment cohorts: gemigliptin monotherapy group, gemigliptin + metformin therapy group, gemigliptin + sulfonylurea therapy group, gemigliptin + sulfonylurea + metformin therapy group, gemigliptin + insulin ± other diabetic medications therapy group, and gemigliptin + other diabetic medications therapy group. These treatment cohorts were classified based on treatments received at the time of baseline, and treatment changes during the study were not considered. The chi-square test or Fisher’s exact test was performed to examine the differences in incidence among the treatment cohorts.

Statistical analyses were performed using SAS version 9.4 (SAS Institute, Inc., Cary, NC).

## Results

### Patient disposition and baseline characteristics

**Figure 1:**
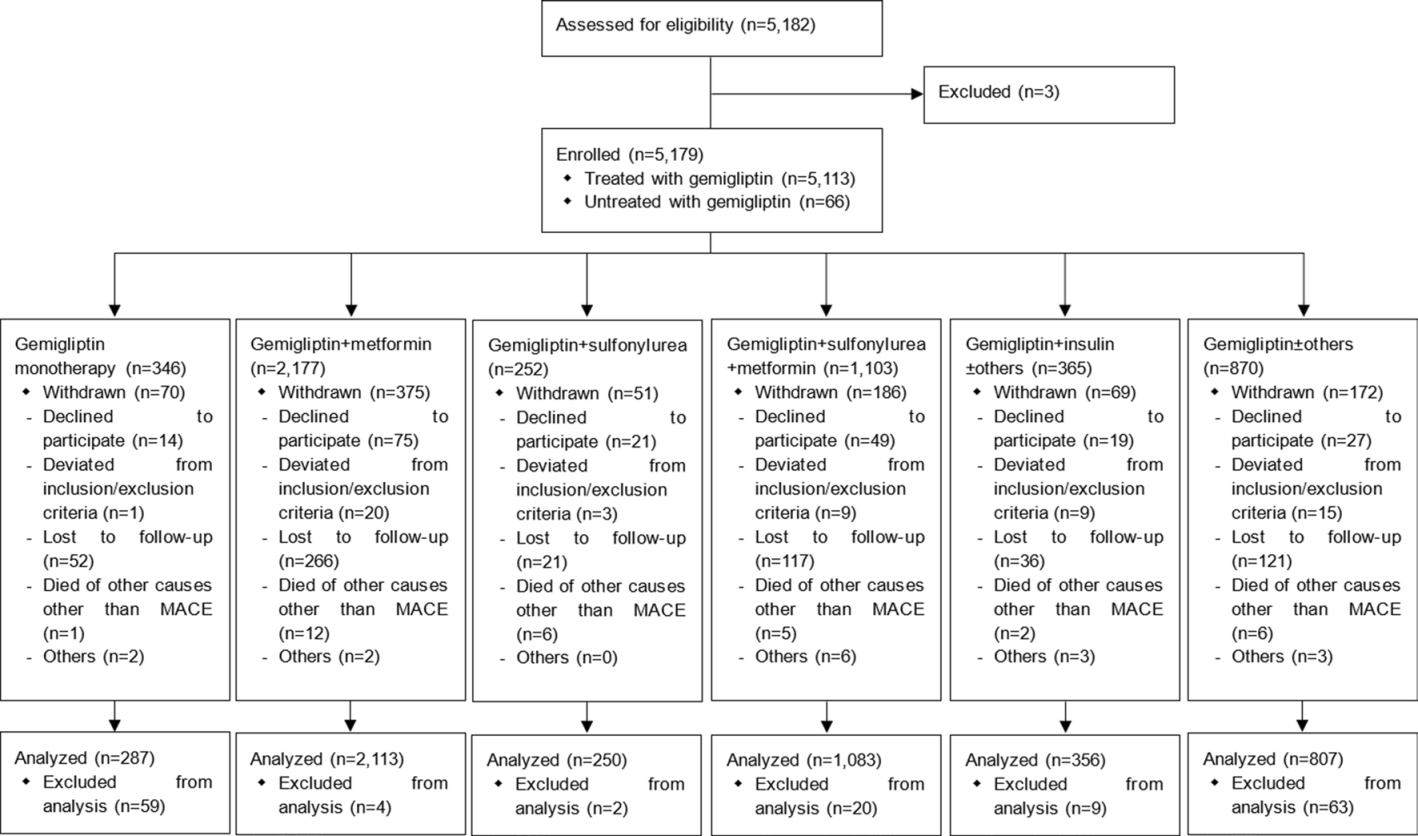
Patient disposition, MACE, major adverse cardiovascular event.

A total of 5182 patients with T2DM were screened, 5,179 were enrolled in the study, and 5113 were treated with gemigliptin (Fig. 1). The majority of the patients (93.16%) received a dose of 50 mg of gemigliptin. In terms of treatment cohort, 346 patients received gemigliptin monotherapy (Gemi Mono group), 2177 received gemigliptin and metformin (Gemi + Met group), 252 received gemigliptin and sulfonylurea (Gemi + SU group), 1103 received gemigliptin, sulfonylurea and metformin (Gemi + SU + Met group), 365 received gemigliptin and insulin and/or other diabetic medications (Gemi + INS ± Others group), and 870 received gemigliptin and other diabetic medications (Gemi + Others group). The mean (standard deviation [SD]) duration of participation in the study was 2.47 (1.03) years, and the maximum period was 4.35 years. A total of 4896 patients who received gemigliptin at least once and were followed up more than once were included in the analysis.

Baseline characteristics are summarized in Table 1. The mean (SD) age of participants was 59.65 (11.82) years and males accounted for 54.21% of the group. The mean (SD) BMI was 25.33 (3.58) kg/m^2^ and the mean (SD) duration of diabetes was 6.05 (6.83) years. In terms of treatment cohort, the mean duration of diabetes was shortest in the Gemi Mono group (1.96 years) and longest in the Gemi + INS ± Others group (12.42 years). The mean (SD) CV risk calculated by the Framingham risk score^20^ was 22.04% (8.48%) and approximately three quarters of the patients had a CV risk > 15%. At baseline, the mean (SD) HbA1c was 8.29% (1.65%). Regarding concurrent diseases, 51.57% of the patients had hypertension, 42.95% had dyslipidemia, and 11.85% had cardiac disorders. As each treatment was determined at the discretion of the investigator taking the patient’s underlying disease and characteristics into consideration, there were significant differences in most of the baseline characteristics among the treatment subgroups.

**Table 1:** Baseline characteristics.

	Gemi Mono (N = 287)	Gemi + Met (N = 2113)	Gemi + SU (N = 250)	Gemi + SU + Met (N = 1083)	Gemi + INS ± others (N = 356)	Gemi + others (N = 807)	Total (N = 4896)
Age (years), n	287	2113	250	1083	356	807	4896
Mean (SD)	56.38 (12.03)	58.78 (11.70)	65.44 (11.09)	61.01 (11.46)	58.79 (12.01)	59.86 (11.88)	59.65 (11.82)
Sex, n (%)							
Male	171 (59.58)	1122 (53.10)	139 (55.60)	616 (56.88)	166 (46.63)	440 (54.52)	2654 (54.21)
Female	116 (40.42)	991 (46.90)	111 (44.40)	467 (43.12)	190 (53.37)	367 (45.48)	2242 (45.79)
Weight (kg), n	177	1595	210	821	324	656	3783
Mean (SD)	66.47 (11.19)	67.65 (12.15)	65.94 (11.71)	66.83 (11.63)	66.96 (12.90)	66.74 (13.20)	67.11 (12.23)
BMI (kg/m^2^), n	175	1516	204	790	299	636	3620
Mean (SD)	24.87 (3.40)	25.50 (3.59)	25.15 (3.60)	25.31 (3.42)	25.48 (3.79)	25.10 (3.67)	25.33 (3.58)
Waist (cm), n	110	808	146	486	189	379	2118
Mean (SD)	86.29 (8.91)	88.51 (9.41)	86.49 (10.56)	87.43 (9.81)	88.31 (10.30)	86.42 (10.59)	87.61 (9.88)
Duration of diabetes (years), n	285	2051	249	1063	351	796	4795
Mean (SD)	1.96 (4.03)	3.91 (5.06)	7.70 (6.93)	8.50 (6.98)	12.42 (8.96)	6.45 (7.02)	6.05 (6.83)
Min, Max	0.00, 26.00	0.00, 39.00	0.00, 39.00	0.00, 54.00	0.00, 39.00	0.00, 54.00	0.00, 54.00
Diabetic medications at enrollment, n (%)							
Yes	1 (0.35)	1704 (80.64)	238 (95.20)	1045 (96.49)	335 (94.10)	724 (89.71)	4047 (82.66)
No	286 (99.65)	409 (19.36)	12 (4.80)	38 (3.51)	21 (5.90)	83 (10.29)	849 (17.34)
Smoking at enrollment, n (%)							
Yes	54 (18.82)	417 (19.73)	56 (22.40)	249 (22.99)	59 (16.57)	166 (20.57)	1001 (20.45)
No	233 (81.18)	1696 (80.27)	194 (77.60)	834 (77.01)	297 (83.43)	641 (79.43)	3895 (79.55)
Cardiovascular risk* (%), n	214	1462	176	767	219	548	3386
Mean (SD)	21.51 (8.62)	21.33 (8.58)	25.77 (6.88)	22.88 (8.18)	20.55 (9.23)	22.36 (8.29)	22.04 (8.48)
Min, Max	3.30, 30.00	1.20, 30.00	5.30, 30.00	2.40, 30.00	1.70, 30.00	2.80, 30.00	1.20, 30.00
HbA1c (%), n	246	1813	208	923	299	655	4144
Mean (SD)	8.54 (1.95)	8.00 (1.51)	8.03 (1.41)	8.64 (1.57)	9.24 (1.86)	8.15 (1.70)	8.29 (1.65)
Min, Max	5.40, 19.10	5.00, 18.50	5.00, 13.10	5.40, 16.10	5.00, 16.80	4.70, 15.70	4.70, 19.10
Total Cholesterol (mg/dL), n	213	1420	180	733	231	535	3312
Mean (SD)	189.84 (45.34)	186.64 (50.74)	183.50 (53.48)	173.99 (45.16)	168.76 (47.72)	177.65 (47.97)	181.18 (49.11)
Min, Max	97.80, 399.00	84.00, 528.00	42.00, 420.00	67.20, 374.00	76.00, 473.00	27.00, 552.00	27.00, 552.00
HDL Cholesterol (mg/dL), n	174	1257	149	634	210	448	2872
Mean (SD)	49.99 (14.79)	49.87 (13.79)	49.49 (17.11)	47.88 (14.19)	48.09 (14.53)	48.34 (14.46)	49.05 (14.30)
Min, Max	22.00, 136.00	15.00, 167.00	18.00, 144.00	15.00, 167.00	26.00, 130.00	7.00, 148.00	7.00, 167.00
Concurrent diseases, n (%)							
Hypertension	103 (35.89)	1030 (48.75)	157 (62.80)	605 (55.86)	194 (54.49)	436 (54.03)	2525 (51.57)
Dyslipidemia	45 (15.68)	926 (43.82)	100 (40.00)	504 (46.54)	166 (46.63)	362 (44.86)	2103 (42.95)
Cardiac disorders	19 (6.62)	216 (10.22)	47 (18.80)	146 (13.48)	45 (12.64)	107 (13.26)	580 (11.85)
Concomitant medications except antidiabetics, n (%)							
Lipid modifying agents	58 (20.21)	1147 (54.28)	132 (52.8)	639 (59)	249 (69.94)	489 (60.59)	2714 (55.43)
Angiotensin II antagonists	76 (26.48)	824 (39)	134 (53.6)	488 (45.06)	151 (42.42)	339 (42.01)	2012 (41.09)
Selective calcium channel blockers	33 (11.5)	308 (14.58)	56 (22.4)	180 (16.62)	71 (19.94)	146 (18.09)	794 (16.22)
Beta blocking agents	25 (8.71)	224 (10.6)	39 (15.6)	158 (14.59)	48 (13.48)	106 (13.14)	600 (12.25)
Diuretics	11 (3.83)	114 (5.40)	30 (12.00)	80 (7.39)	54 (15.17)	70 (8.67)	359 (7.33)
ACE inhibitors	3 (1.05)	50 (2.37)	8 (3.20)	48 (4.43)	23 (6.46)	32 (3.97)	164 (3.35)
Gemigliptin treatment duration (years), n	287	2113	250	1083	356	807	4896
Mean (SD)	2.09 (1.15)	2.46 (1.01)	2.50 (1.00)	2.54 (1.03)	1.85 (0.83)	2.47 (1.06)	2.41 (1.04)
Min, Max	0.00, 4.16	0.00, 4.34	0.00, 4.09	0.00, 4.10	0.00, 4.04	0.00, 4.35	0.00, 4.35

SD, standard deviation; Min, minimum; Max, maximum; HbA1c, glycated hemoglobin.

*Cardiovascular risk was calculated according to the Framingham risk score calculation method.

### Primary composite MACE

**Figure 2:**
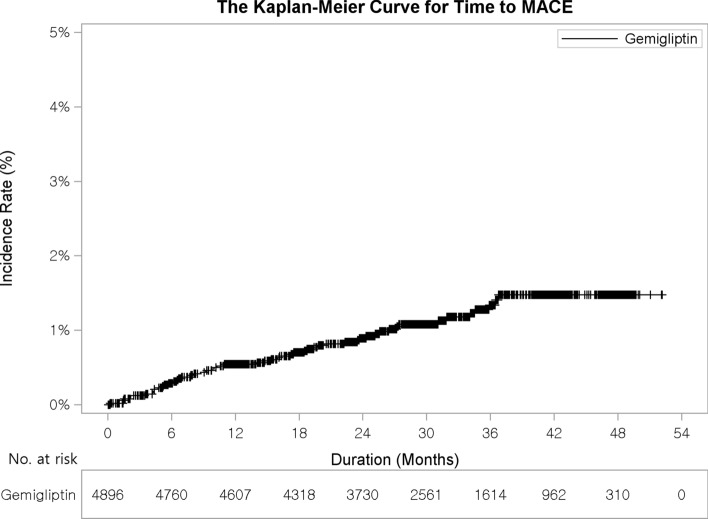
Time to occurrence of the primary composite MACE, MACE, major adverse cardiovascular event.

Overall, the primary composite MACE occurred in 26 patients within 12 months (estimated incidence 0.49%, 95% CI 0.29–0.69%) and in 54 patients within 54 months (estimated incidence 1.35%, 95% CI: 0.92–1.77%) (Fig. 2, Supplementary Table S1). Because the upper limit of the 95% CI of the annual incidence was < 2%, it was confirmed that gemigliptin did not significantly increase the incidence of the primary composite MACE. In the subgroup analysis based on treatment cohort, the estimated incidence in the Gemi Mono group at 54 months was the lowest at 0.04%, whereas that in the Gemi + SU group was the highest at 1.69% (Supplementary Table S1 and Fig. S1). Overall, for each component of the primary composite MACE, CV death occurred in two patients within 54 months, nonfatal myocardial infarction occurred in 25 patients, and nonfatal ischemic stroke occurred in 30 patients (Supplementary Table S2). The incidence of the composite MACE was 1.10% (4.7 events per 1000 person-years) during the study period and differences in incidence among treatment cohorts were not significant (*p* = 0.3221) (Table 2).

**Table 2:** Incidence of the primary composite MACE.

	Gemi Mono (N = 287)	Gemi + Met (N = 2113)	Gemi + SU (N = 250)	Gemi + SU + Met (N = 1083)	Gemi + INS ± others (N = 356)	Gemi + others (N = 807)	Total (N = 4896)
Composite MACE							
Incidence, n (%)	2 (0.70)	19 (0.90)	6 (2.40)	14 (1.29)	5 (1.40)	8 (0.99)	54 (1.10)
95% confidence interval	(0.00, 1.66)	(0.50, 1.30)	(0.50, 4.30)	(0.62, 1.97)	(0.18, 2.63)	(0.31, 1.67)	(0.81, 1.40)
Annualized incidence rate (per 1000 person-years)	3.3	3.5	12.1	5.1	7.9	4.2	4.7
* p *value [1]							0.3221 (c)
Hazard ratio between treatment cohorts [2]							
Hazard ratio (95% confidence interval)		1.00 (0.23, 4.33)	1.95 (0.38, 9.91)	1.16 (0.26, 5.26)	1.52 (0.27, 8.43)	0.98 (0.20, 4.68)	
Cardiovascular death							
Incidence, n (%)	1 (0.35)	1 (0.05)	0 (0.00)	0 (0.00)	0 (0.00)	0 (0.00)	2 (0.04)
95% confidence interval	(0.00, 1.03)	(0.00, 0.14)	(0.00, 0.00)	(0.00, 0.00)	(0.00, 0.00)	(0.00, 0.00)	(0.00, 0.10)
Annualized incidence rate (per 1000 person-years)	1.6	0.2	-	-	-	-	0.2
* p *value [1]							0.3448 (f)
Hazard ratio between treatment cohorts [2]							
Hazard ratio (95% confidence interval)		0.21 (0.01, 3.43)	0.00 (0.00, NC)	0.00 (0.00, NC)	0.00 (0.00, NC)	0.00 (0.00, NC)	
Nonfatal myocardial infarction							
Incidence, n (%)	1 (0.35)	7 (0.33)	4 (1.60)	7 (0.65)	3 (0.84)	3 (0.37)	25 (0.51)
95% confidence interval	(0.00, 1.03)	(0.09, 0.58)	(0.04, 3.16)	(0.17, 1.12)	(0.00, 1.79)	(0.00, 0.79)	(0.31, 0.71)
Annualized incidence rate (per 1000 person-years)	1.6	1.3	7.6	2.4	5.3	1.9	2.2
* p *value [1]							0.1152 (f)
Hazard ratio between treatment cohorts [2]							
Hazard ratio (95% confidence interval)		0.71 (0.09, 5.85)	2.19 (0.23, 20.44)	1.08 (0.13, 9.20)	1.72 (0.16, 18.56)	0.69 (0.07, 6.83)	
Nonfatal ischemic stroke							
Incidence, n (%)	1 (0.35)	12 (0.57)	3 (1.20)	7 (0.65)	2 (0.56)	5 (0.62)	30 (0.61)
95% confidence interval	(0.00, 1.03)	(0.25, 0.89)	(0.00, 2.55)	(0.17, 1.12)	(0.00, 1.34)	(0.08, 1.16)	(0.39, 0.83)
Annualized incidence rate (per 1000 person-years)	1.6	2.2	4.5	2.7	2.6	2.3	2.5
* p *value [1]							0.8561 (f)
Hazard ratio between treatment cohorts [2]							
Hazard ratio (95% confidence interval)		1.29 (0.17, 9.94)	2.14 (0.21, 21.31)	1.20 (0.14, 10.11)	1.25 (0.10, 14.97)	1.25 (0.14, 10.94)	

MACE, major adverse cardiovascular event; NC, not calculated.

[1] Difference among the treatment cohorts [Chi-square test (c) or Fisher’s exact test (f)].

[2] Hazard ratio is predicted by Cox regression (Proportional Hazard Model) with age, sex, smoking and duration of diabetes as covariates (Gemigliptin monotherapy group [Reference] vs. Combination therapy group).

### All-cause mortality and other cardiovascular events

**Figure 3:**
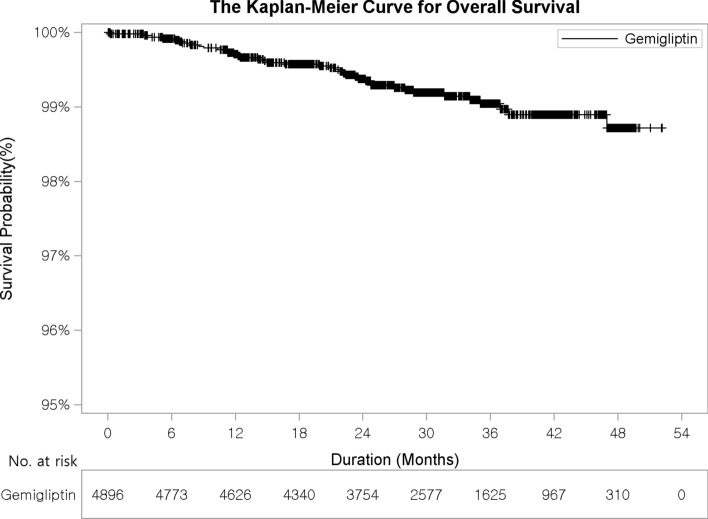
The Kaplan–Meier curve for overall survival.

Overall, the incidence of all-cause mortality was 0.82% (3.2 events per 1,000 person-years) and differences in incidence among treatment cohorts were not significant (*p* = 0.0762). The Kaplan–Meier curve for overall survival is presented in Fig. 3. The incidence of HF was 0.22% (1.1 events per 1000 person-years), the incidence of peripheral vascular disease was 0.02% (0.1 events per 1000 person-years), the incidence of unstable angina pectoris was 0.29% (1.2 events per 1000 person-years), and there were no cases of hospitalization due to revascularization (Supplementary Table S3).

### Malignancies and other adverse events

The incidence of overall adverse events was 28.70% (231.7 events per 1000 person-years) and most adverse events were mild to moderate in severity. The most frequently reported adverse event was hyperlipidemia (2.25%, 8.9 events per 1000 person-years). The incidence of adverse events of special interest (any malignancies, arthralgia, hypersensitivity, or severe skin reactions) was less than 2%, and there were no cases of pancreatitis, increased blood amylase, increased lipase, or bacteriuria (Table 3). Of the adverse events of special interest, only three cases of arthralgia and one case of malignancy were reported as adverse drug reactions, and only four cases of MACE were reported as adverse drug reactions. Most adverse drug reactions were resolved during the study period.

**Table 3:** Adverse events.

	Gemi Mono (N = 287)	Gemi + Met (N = 2113)	Gemi + SU (N = 250)	Gemi + SU + Met (N = 1083)	Gemi + INS ± others (N = 356)	Gemi + others (N = 807)	Total (N = 4896)
All adverse events	41 (14.29)/143.2	538 (25.46)/190.3	79 (31.60)/239.2	326 (30.10)/215.7	164 (46.07)/542.2	257 (31.85)/272.1	1405 (28.70)/231.7
Adverse events of special interest							
Any malignancies	2 (0.70)/4.9	26 (1.23)/4.8	6 (2.40)/10.6	13 (1.20)/4.4	4 (1.12)/5.3	10 (1.24)/5.6	61 (1.25)/5.2
Arthralgia	1 (0.35)/1.6	9 (0.43)/1.6	2 (0.80)/3.0	6 (0.55)/2.4	4 (1.12)/6.6	7 (0.87)/3.3	29 (0.59)/2.5
Hypersensitivity	0	0	1 (0.40)/1.5	1 (0.09)/0.3	0	0	2 (0.04)/0.2
Severe skin reactions	0	0	0	0	1 (0.28)/1.3	0	1 (0.02)/0.1
Pancreatitis	0	0	0	0	0	0	0
Blood amylase increased	0	0	0	0	0	0	0
Lipase increased	0	0	0	0	0	0	0
Bacteriuria	0	0	0	0	0	0	0
Adverse drug reactions	2 (0.70)/4.9	45 (2.13)/9.7	7 (2.80)/15.1	34 (3.14)/17.4	15 (4.21)/24.9	29 (3.59)/16.3	132 (2.70)/13.6
Hypoglycaemia	1 (0.35)/1.6	7 (0.33)/1.5	2 (0.80)/3.0	6 (0.55)/2.7	7 (1.97)/11.8	10 (1.24)/4.7	33 (0.67)/3.0
Headache	0	3 (0.14)/0.5	0	3 (0.28)/1.0	0	3 (0.37)/1.4	9 (0.18)/0.7
Any malignancies	0	0	0	1 (0.09)/0.3	0	0	1 (0.02)/0.1
Arthralgia	0	1 (0.05)/0.2	0	1 (0.09)/0.3	0	1 (0.12)/0.5	3 (0.06)/0.2
Carotid artery stenosis*	0	1 (0.05)/0.2	0	0	0	0	1 (0.02)/0.1
Cerebral infarction*	0	0	1 (0.40)/1.5	0	0	0	1 (0.02)/0.1
Cerebrovascular accident*	0	0	0	1 (0.09)/0.3	0	0	1 (0.02)/0.1
Transient ischaemic attack*	0	0	0	0	0	1 (0.12)/0.5	1 (0.02)/0.1
Serious adverse events	16 (5.57)/39.0	120 (5.68)/31.8	33 (13.20)/74.2	98 (9.05)/45.2	59 (16.57)/141.8	72 (8.92)/59.5	398 (8.13)/48.9
Serious adverse drug reactions	0	3 (0.14)/0.7	4 (1.60)/6.1	5 (0.46)/1.7	3 (0.84)/3.9	3 (0.37)/1.4	18 (0.37)/1.5
Adverse events leading to discontinuation of study drug	2 (0.70)/4.9	32 (1.51)/6.8	8 (3.20)/13.6	22 (2.03)/8.2	10 (2.81)/13.1	26 (3.22)/15.8	100 (2.04)/9.3
Hypoglycaemia	0	4 (0.19)/0.7	2 (0.80)/3.0	2 (0.18)/0.7	0	2 (0.25)/0.9	10 (0.20)/0.8
Any malignancies	0	2 (0.09)/0.4	0	2 (0.18)/0.7	0	3 (0.37)/1.4	7 (0.14)/0.6
Arthralgia	0	0	0	0	0	1 (0.12)/0.5	1 (0.02)/0.1
Acute myocardial infarction*	0	1 (0.05)/0.2	0	0	0	1 (0.12)/0.5	2 (0.04)/0.2
Cerebral infarction*	0	0	0	1 (0.09)/0.3	0	0	1 (0.02)/0.1
Myocardial infarction*	1 (0.35)/1.6	0	0	0	0	0	1 (0.02)/0.1
Adverse events leading to death	2 (0.70)/3.3	14 (0.66)/2.7	6 (2.40)/9.1	7 (0.65)/2.4	2 (0.56)/2.6	9 (1.12)/4.7	40 (0.82)/3.3
Any malignancies	1 (0.35)/1.6	3 (0.14)/0.5	0	1 (0.09)/0.3	0	0	5 (0.10)/0.4
Acute myocardial infarction*	0	1 (0.05)/0.2	0	0	0	0	1 (0.02)/0.1
Myocardial infarction*	1 (0.35)/1.6	0	0	0	0	0	1 (0.02)/0.1

Data are the number of patients (%)/events per 1000 person-years.

*These adverse events were reported as major adverse cardiovascular event.

### HbA1c changes and other results

**Figure 4:**
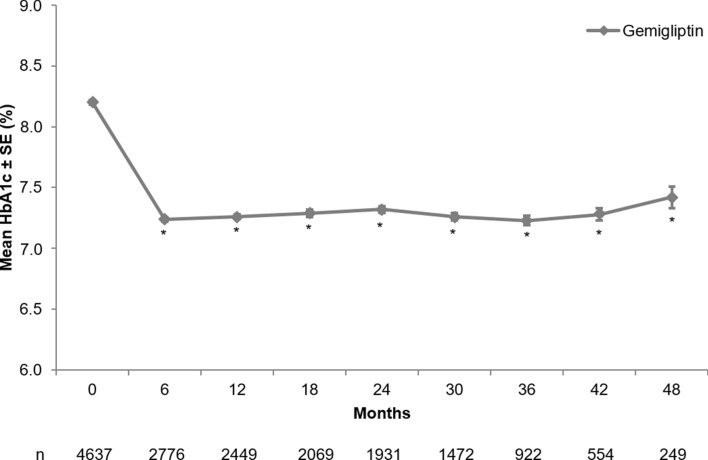
HbA1c level over time. HbA1c, glycated hemoglobin; SE, standard error. * The change in HbA1c from baseline was significant (*p* < 0.0001). *p* value was obtained from Wilcoxon signed rank test.

After 6 months of gemigliptin administration, HbA1c levels were reduced by 0.94% compared to the baseline (*p* < 0.0001). The mean change (SD) in HbA1c levels relative to the baseline at 24 months was − 0.83% (1.61%), and reduction in HbA1c levels was also observed at 48 months (mean change − 0.40%; Fig. 4).

After participating in this study, the treatments for 27.41% of the patients were adjusted―e.g., gemigliptin was discontinued and/or other antidiabetic agents were administered. Additionally, 22.39% of the patients received other antidiabetic agents after administration of gemigliptin, and of the antidiabetic agents, sulfonylureas (38.87%) and metformin (25.18%) were the most commonly administered. The gemigliptin dose was also changed or discontinued for 8.70% of the patients.

## Discussion

In this observational study, the estimated incidence of MACE at 12 months of follow-up, which was a primary endpoint, was 0.49%, and that at 54 months was 1.35%. In addition, the incidence of MACE at every time point in 6-month interval was all < 2%, which was statistically significant. Therefore, the use of gemigliptin (as monotherapy or part of combination therapy) did not significantly increase MACE incidence.

DPP-4 inhibitors which are drugs that regulate blood glucose by increasing insulin secretion and inhibiting the degradation of GLP-1 by reacting with blood glucose have strong anti-hypoglycemic effects^6^. DPP-4 inhibitors had been expected to confer beneficial effects on CV disease (CVD) due to pleiotropic effects as they show positive effects on CV risk factors in T2DM patients, such as weight loss, reduced blood pressure, improved postprandial dyslipidemia, and reduced inflammation^21^. In addition, DPP-4 inhibitors may have a direct protective effect against CVDs, which may be mediated by the inhibition of endoplasmic reticulum (ER) stress in cardiomyocytes, vascular calcification, and vascular remodeling^22^. Gemigliptin effectively inhibited ER stress-induced apoptosis and inflammation via the *Akt*/protein kinase RNA-like endoplasmic reticulum kinase (PERK)/C-EBP homologous protein (CHOP) and inositol-requiring enzyme 1 α (IRE1α)/c-Jun N-terminal kinase (JNK)-p38 pathways in H9C2 cardiomyocytes in vitro^22^. In addition, gemigliptin attenuated vascular calcification in vitro and in vivo and osteogenic transdifferentiation of vascular smooth muscle cells (VSMCs) by reducing PiT-1 expression, attenuating phosphate-induced oxidative stress, phospho-AKT/PI3K signaling, and Wnt signaling^23^. Although DPP4 inhibitors have been considered to have beneficial effects on CVD from experimental studies, there was no evidence of beneficial effects on CVD with DPP4 inhibitors from large-scale CV outcome studies^14–19^.

In the results of the subgroup analyses, the incidence of MACE was lowest in the Gemi Mono and Gemi + Met groups and highest in the Gemi + SU group. The differences of these findings among the treatment subgroups are likely primarily due to differences of the baseline characteristics among subgroups. On the other hand, we can assume the reason from the studies that revealing that fewer CV events occurred in patients treated with metformin compared to patients treated with sulfonylurea or a combination of metformin and sulfonylurea^24^.

The incidence of MACE was 8.4% in a large-scale CV outcome study of sitagliptin^14^, another DPP-4 inhibitor, 11.3% in a study of alogliptin^16^, 12.4% in a study of linagliptin^17^, and 7.3% in a study of saxagliptin^15^, all of which are values higher that reported in this study for gemigliptin. The incidence of MACE in this study was lower upon simple comparison with the results of the previous studies outlined above. However, while these previous reports presented results of randomized placebo-controlled studies, the present study had different design as a non-interventional study, and there were differences in the methods of evaluation, adjudication and analysis of MACE. In addition, considering the different baseline characteristics of the subjects, particularly the younger age of subjects enrolled in this study, and the lower rate of subjects with a medical history of CVD compared to that of the cohorts in previous studies, it was determined that the incidence of MACE was lower than in previous studies as our subjects exhibited relatively fewer CV risk factors.

In a retrospective analysis with data from several nationwide registries in Denmark, sitagliptin monotherapy was not associated with any significant increase in risk of a composite endpoint of stroke, acute myocardial infarction and all-cause mortality compared with metformin monotherapy^25^. In the Danish retrospective study, subjects using sitagliptin had a relatively short duration of monotherapy (0.9 year) and the incidence of the composite endpoint was 5.0%. Another retrospective analysis of nationwide data from Taiwan’s National Health Insurance Research Database demonstrated that DPP-4 inhibitors (sitagliptin, vildagliptin and saxagliptin), compared with sulfonylureas, were associated with lower risks of all-cause death and MACEs (ischemic stroke and myocardial infarction) as add-ons to metformin therapy^26^. In this retrospective cohort study, 209 MACEs occurred during a 3.3-year follow-up period in patients using DPP-4 inhibitors (10.4 events per 1000 person-years). As prospective observational study, this current study provided additional information of CV safety in the gemigliptin as one of new DPP-4 inhibitors (MACE, 4.7 events per 1000 person-years).

The incidences of each component of MACE and other CV events (HF, hospitalization due to revascularization, peripheral vascular disease, unstable angina pectoris) were low with values < 1%. Previous large-scale CV outcome studies of different DPP-4 inhibitors did not provide clear data regarding HF risk^27^. However, the potential for increased risk of HF with DPP-4 inhibitors was reported in the saxagliptin study, in which patients with T2DM and either a history of CVD or multiple CV risk factors were randomized to receive saxagliptin or placebo^15^. We also evaluated the incidence of HF, which was 0.22% for the whole patients and ≤ 0.50% for all subgroups in this study.

In this study, the incidence of all-cause mortality was 0.82%. According to the analysis of National Health Insurance data^27^, the all-cause mortality risk was lower for patients receiving metformin and DPP-4 inhibitors compared to patients taking metformin and sulfonylurea (hazard ratio [HR] 0.84, 95% CI 0.66–1.07)^28^. The risk of all-cause mortality was compared among five types of DPP-4 inhibitors, and CV risk was shown to be lower for gemigliptin compared to sitagliptin (HR 0.84, 95% CI 0.80–0.88)^29^.

DPP-4 inhibitors are relatively well-tolerated and have fewer side effects compared to other antidiabetic agents. However, they must be prescribed carefully, as adverse events, such as angioedema, anaphylaxis and Stevens-Johnson syndrome, have been reported albeit rarely^30^. In addition, a previous study showing increases in number of pancreatic duct cells in response to incretin-based treatment suggested that DPP-4 inhibitors may be associated with pancreas-related safety issues, such as pancreatitis and pancreatic cancer^31^. In large-scale CV outcome trials, sitagliptin was associated with incidence of 0.3% for pancreatitis and 0.1% for pancreatic cancer, and saxagliptin was associated with incidence of 0.3% and 0.06%, respectively^14,15^. However, in this large-scale prospective observational study, pancreatitis and increased blood amylase and lipase levels were not reported. The incidence of all types of cancer was 1.25%, which was lower than the reported incidence for other DPP-4 inhibitors (3.7% for sitagliptin^14^, 3.3% for linagliptin^17^, and 3.9% for saxagliptin^15^). One case of pancreatic cancer was reported in this study. However, it was not considered to be related to gemigliptin as the patient had a medical history of acute pancreatitis prior to enrollment in this study and the duration of gemigliptin treatment was short (less than 1 month). In addition, gemigliptin is considered to be safe for long-term use as the incidences of other events such as arthralgia, hypersensitivity, and severe skin reactions were low.

This study had limitations. This non-interventional observational study did not involve comparisons with a placebo or other DPP-4 inhibitors. It also had the limitation that it was difficult to collect accurate data via self-reporting of MACE from the patients. In addition, the methods of evaluation, adjudication and analysis of MACE were different and patients with relatively low CV risk (younger age, lower rate of concurrent hypertension and history of CVDs, and shorter duration of diabetes) were enrolled in this study compared to previous large-scale CV outcome studies conducted as placebo-controlled randomized studies, and these factors may have influenced the results of this study, such as the incidences of MACE and other adverse events. The subgroup analyses of this study were performed for exploratory purposes based on the treatment cohorts, but this also had some limitations. Participants were allowed to change or discontinue their treatment during the study period at the discretion of the investigator, but treatment changes were not considered in the MACE analysis. In addition, some of the baseline characteristics, such as the history of CVD, had significant differences among the treatment subgroups, but were not included as covariates in a Cox regression model, and HbA1c level was also not adjusted in the MACE analysis due to insufficient data collection. Despite these limitations, this study yielded meaningful information regarding the safety of DPP-4 inhibitors through a large-scale long-term prospective cohort study with a wide range of T2DM patients who used gemigliptin alone or as combination therapy.

In conclusion, this study showed that gemigliptin use was associated with low incidences of the primary composite MACE, all-cause mortality, and other adverse events in patients with T2DM in real practice. In addition, it was also confirmed that combination therapy of gemigliptin with other antidiabetic agents (metformin, insulin and other agents except sulfonylurea) did not increase the incidence of CV events. Thus, the clinical use of gemigliptin may safe, and it also may not increase CV risk in real practice.

## Supplementary information

Supplementary Information

## Data Availability

The datasets analyzed during the current study are available from the corresponding author on reasonable request.
