# Supplementary material for: A prospective cohort study on effects of gemigliptin on cardiovascular outcomes in patients with type 2 diabetes (OPTIMUS study)
Source: Sci Rep. 2020 Nov 4;10:19033. doi: 10.1038/s41598-020-75594-5 (PMC7642439; doi:10.1038/s41598-020-75594-5)
Supplement: Supplementary file 1 — Supplementary Information [file 41598_2020_75594_MOESM1_ESM.docx]

**A prospective cohort study on effects of gemigliptin on cardiovascular outcomes in patients with type 2 diabetes (OPTIMUS study)**

Sang Soo Kim, Eun Heui Kim, Dong Jun Kim, Young Sik Choi, Chang Won Lee, Bon Jeong Ku, Kwang Soo Cha, Kee Ho Song, Dae Kyeong Kim and In Joo Kim

Supplementary Information

Table of Content

[**Table S1. Time to occurrence of the primary composite MACE** 3](#_Toc24708320)

[**Table S2. Estimates of the incidence of the each component of the primary composite MACE at 54 months** 6](#_Toc24708321)

[**Table S3. Incidence of other cardiovascular events** 8](#_Toc24708322)

[**Figure S1. Time to occurrence of the primary composite MACE by treatment cohort** 10](#_Toc24708323)

# **Table S1. Time to occurrence of the primary composite MACE**

|  | Gemi Mono (N=287) | Gemi + Met (N=2113) | Gemi + SU (N=250) | Gemi + SU + Met (N=1083) | Gemi + INS ± others (N=356) | Gemi + others (N=807) | Total (N=4896) |
| --- | --- | --- | --- | --- | --- | --- | --- |
| 6 months |  |  |  |  |  |  |  |
| Number of subjects with MACEs | 0 | 6 | 1 | 2 | 2 | 3 | 14 |
| Incidence rate (%) [1] | - | 0.21 | 0.26 | 0.16 | 0.42 | 0.28 | 0.26 |
| 95% confidence interval | [-, -] | (0.02, 0.39) | (0.00, 0.80) | (0.00, 0.39) | (0.00, 1.07) | (0.00, 0.62) | (0.12, 0.40) |
| Incidence rate (%) [2] |  |  |  |  |  |  | 0.26 |
| 95% confidence interval |  |  |  |  |  |  | (0.12, 0.40) |
| 12 months |  |  |  |  |  |  |  |
| Number of subjects with MACEs | 0 | 9 | 4 | 6 | 3 | 4 | 26 |
| Incidence rate (%) [1] | - | 0.31 | 1.03 | 0.49 | 0.63 | 0.37 | 0.49 |
| 95% confidence interval | [-, -] | (0.07, 0.55) | (0.00, 2.38) | (0.07, 0.91) | (0.00, 1.49) | (0.00, 0.78) | (0.29, 0.69) |
| Incidence rate (%) [2] |  |  |  |  |  |  | 0.48 |
| 95% confidence interval |  |  |  |  |  |  | (0.29, 0.68) |
| 18 months |  |  |  |  |  |  |  |
| Number of subjects with MACEs | 0 | 13 | 5 | 6 | 3 | 6 | 33 |
| Incidence rate (%) [1] | - | 0.46 | 1.30 | 0.49 | 0.63 | 0.57 | 0.63 |
| 95% confidence interval | [-, -] | (0.15, 0.78) | (0.00, 2.89) | (0.07, 0.91) | (0.00, 1.49) | (0.02, 1.12) | (0.40, 0.87) |
| Incidence rate (%) [2] |  |  |  |  |  |  | 0.62 |
| 95% confidence interval |  |  |  |  |  |  | (0.39, 0.85) |
| 24 months |  |  |  |  |  |  |  |
| Number of subjects with MACEs | 2 | 13 | 5 | 11 | 4 | 6 | 41 |
| Incidence rate (%) [1] | 0.04 | 0.46 | 1.30 | 0.95 | 0.87 | 0.57 | 0.81 |
| 95% confidence interval | (0.00, 41.14) | (0.15, 0.78) | (0.00, 2.89) | (0.32, 1.57) | (0.00, 1.94) | (0.02, 1.12) | (0.54, 1.08) |
| Incidence rate (%) [2] |  |  |  |  |  |  | 0.79 |
| 95% confidence interval |  |  |  |  |  |  | (0.53, 1.06) |
| 30 months |  |  |  |  |  |  |  |
| Number of subjects with MACEs | 2 | 15 | 6 | 11 | 5 | 8 | 47 |
| Incidence rate (%) [1] | 0.04 | 0.56 | 1.69 | 0.95 | 1.30 | 0.86 | 0.98 |
| 95% confidence interval | (0.00, 41.14) | (0.20, 0.93) | (0.00, 3.66) | (0.32, 1.57) | (0.00, 2.84) | (0.10, 1.62) | (0.67, 1.29) |
| Incidence rate (%) [2] |  |  |  |  |  |  | 0.96 |
| 95% confidence interval |  |  |  |  |  |  | (0.65, 1.27) |
| 36 months |  |  |  |  |  |  |  |
| Number of subjects with MACEs | 2 | 18 | 6 | 13 | 5 | 8 | 52 |
| Incidence rate (%) [1] | 0.04 | 0.83 | 1.69 | 1.29 | 1.30 | 0.86 | 1.21 |
| 95% confidence interval | (0.00, 41.14) | (0.30, 1.35) | (0.00, 3.66) | (0.47, 2.10) | (0.00, 2.84) | (0.10, 1.62) | (0.83, 1.60) |
| Incidence rate (%) [2] |  |  |  |  |  |  | 1.20 |
| 95% confidence interval |  |  |  |  |  |  | (0.82, 1.58) |
| 42 months |  |  |  |  |  |  |  |
| Number of subjects with MACEs | 2 | 19 | 6 | 14 | 5 | 8 | 54 |
| Incidence rate (%) [1] | 0.04 | 0.94 | 1.69 | 1.53 | 1.30 | 0.86 | 1.35 |
| 95% confidence interval | (0.00, 41.14) | (0.35, 1.54) | (0.00, 3.66) | (0.57, 2.49) | (0.00, 2.84) | (0.10, 1.62) | (0.92, 1.77) |
| Incidence rate (%) [2] |  |  |  |  |  |  | 1.33 |
| 95% confidence interval |  |  |  |  |  |  | (0.90, 1.76) |
| 48 months |  |  |  |  |  |  |  |
| Number of subjects with MACEs | 2 | 19 | 6 | 14 | 5 | 8 | 54 |
| Incidence rate (%) [1] | 0.04 | 0.94 | 1.69 | 1.53 | 1.30 | 0.86 | 1.35 |
| 95% confidence interval | (0.00, 41.14) | (0.35, 1.54) | (0.00, 3.66) | (0.57, 2.49) | (0.00, 2.84) | (0.10, 1.62) | (0.92, 1.77) |
| Incidence rate (%) [2] |  |  |  |  |  |  | 1.33 |
| 95% confidence interval |  |  |  |  |  |  | (0.90, 1.76) |
| 54 months |  |  |  |  |  |  |  |
| Number of subjects with MACEs | 2 | 19 | 6 | 14 | 5 | 8 | 54 |
| Incidence rate (%) [1] | 0.04 | 0.94 | 1.69 | 1.53 | 1.30 | 0.86 | 1.35 |
| 95% confidence interval | (0.00, 41.14) | (0.35, 1.54) | (0.00, 3.66) | (0.57, 2.49) | (0.00, 2.84) | (0.10, 1.62) | (0.92, 1.77) |
| Incidence rate (%) [2] |  |  |  |  |  |  | 1.33 |
| 95% confidence interval |  |  |  |  |  |  | (0.90, 1.76) |

MACE, major adverse cardiovascular event

[1] Incidence rate by treatment cohort is predicted by Cox regression (Proportional Hazard Model) with age, sex, smoking and duration of diabetes as covariates.

[2] Incidence rate is predicted by Cox regression (Proportional Hazard Model) with treatment cohort, age, sex, smoking and duration of diabetes as covariates.

# **Table S2. Estimates of the incidence of the each component of the primary composite MACE at 54 months**

|  | Gemi Mono (N=287) | Gemi + Met (N=2113) | Gemi + SU (N=250) | Gemi + SU + Met (N=1083) | Gemi + INS ± others (N=356) | Gemi + others (N=807) | Total (N=4896) |
| --- | --- | --- | --- | --- | --- | --- | --- |
| Cardiovascular death |  |  |  |  |  |  |  |
| Number of subjects with cardiovascular death | 1 | 1 | 0 | 0 | 0 | 0 | 2 |
| Incidence rate (%) [1] | 0.0000 | 0.0000 | - | - | - | - | 0.0000 |
| 95% confidence interval | (0.0000, 0.0000) | (0.0000, 0.0000) | [-, -] | [-, -] | [-, -] | [-, -] | (0.0000, 0.0000) |
| Incidence rate (%) [2] |  |  |  |  |  |  | 0.0000 |
| 95% confidence interval |  |  |  |  |  |  | (0.0000, 0.0000) |
| Nonfatal myocardial infarction |  |  |  |  |  |  |  |
| Number of subjects with nonfatal myocardial infarction | 1 | 7 | 4 | 7 | 3 | 3 | 25 |
| Incidence rate (%) [1] | 0.00 | 0.30 | 1.05 | 0.75 | 0.00 | 0.00 | 0.52 |
| 95% confidence interval | (0.00, 0.00) | (0.00, 0.63) | (0.00, 2.66) | (0.00, 1.49) | (0.00, 0.16) | (0.00, 0.00) | (0.24, 0.79) |
| Incidence rate (%) [2] |  |  |  |  |  |  | 0.49 |
| 95% confidence interval |  |  |  |  |  |  | (0.22, 0.77) |
| Nonfatal ischemic stroke |  |  |  |  |  |  |  |
| Number of subjects with nonfatal ischemic stroke | 1 | 12 | 3 | 7 | 2 | 5 | 30 |
| Incidence rate (%) [1] | 0.00 | 0.61 | 0.00 | 0.61 | 0.26 | 0.62 | 0.79 |
| 95% confidence interval | (0.00, 0.02) | (0.12, 1.09) | (0.00, 2.26) | (0.06, 1.17) | (0.00, 1.05) | (0.01, 1.23) | (0.47, 1.11) |
| Incidence rate (%) [2] |  |  |  |  |  |  | 0.78 |
| 95% confidence interval |  |  |  |  |  |  | (0.46, 1.10) |

MACE, major adverse cardiovascular event

[1] Incidence rate by treatment cohort is predicted by Cox regression (Proportional Hazard Model) with age, sex, smoking and duration of diabetes as covariates.

[2] Incidence rate is predicted by Cox regression (Proportional Hazard Model) with treatment cohort, age, sex, smoking and duration of diabetes as covariates.

# **Table S3. Incidence of other cardiovascular events**

|  | Gemi Mono (N=287) | Gemi + Met (N=2113) | Gemi + SU (N=250) | Gemi + SU + Met (N=1083) | Gemi + INS ± others (N=356) | Gemi + others (N=807) | Total (N=4896) |
| --- | --- | --- | --- | --- | --- | --- | --- |
| Heart failure |  |  |  |  |  |  |  |
| Incidence, n (%) | 0 (0.00) | 2 (0.09) | 0 (0.00) | 4 (0.37) | 1 (0.28) | 4 (0.50) | 11 (0.22) |
| 95% confidence interval | (0.00, 0.00) | (0.00, 0.23) | (0.00, 0.00) | (0.01, 0.73) | (0.00, 0.83) | (0.01, 0.98) | (0.09, 0.36) |
| Annualized incidence rate (per 1000 person-years) | - | 0.5 | - | 1.7 | 2.6 | 1.9 | 1.1 |
| *P*-value [1] |  |  |  |  |  |  | 0.2365 |
| Hospitalization due to revascularization |  |  |  |  |  |  |  |
| Incidence, n (%) | 0 (0.00) | 0 (0.00) | 0 (0.00) | 0 (0.00) | 0 (0.00) | 0 (0.00) | 0 (0.00) |
| 95% confidence interval | (0.00, 0.00) | (0.00, 0.00) | (0.00, 0.00) | (0.00, 0.00) | (0.00, 0.00) | (0.00, 0.00) | (0.00, 0.00) |
| Annualized incidence rate (per 1000 person-years) | - | - | - | - | - | - | - |
| *P*-value [1] |  |  |  |  |  |  | NC |
| Peripheral vascular disease |  |  |  |  |  |  |  |
| Incidence, n (%) | 0 (0.00) | 1 (0.05) | 0 (0.00) | 0 (0.00) | 0 (0.00) | 0 (0.00) | 1 (0.02) |
| 95% confidence interval | (0.00, 0.00) | (0.00, 0.14) | (0.00, 0.00) | (0.00, 0.00) | (0.00, 0.00) | (0.00, 0.00) | (0.00, 0.06) |
| Annualized incidence rate (per 1000 person-years) | - | 0.2 | - | - | - | - | 0.1 |
| *P*-value [1] |  |  |  |  |  |  | 1.0000 |
| Unstable angina pectoris |  |  |  |  |  |  |  |
| Incidence, n (%) | 0 (0.00) | 5 (0.24) | 2 (0.80) | 3 (0.28) | 3 (0.84) | 1 (0.12) | 14 (0.29) |
| 95% confidence interval | (0.00, 0.00) | (0.03, 0.44) | (0.00, 1.90) | (0.00, 0.59) | (0.00, 1.79) | (0.00, 0.37) | (0.14, 0.44) |
| Annualized incidence rate (per 1000 person-years) | - | 0.9 | 3.0 | 1.0 | 5.3 | 0.5 | 1.2 |
| *P*-value [1] |  |  |  |  |  |  | 0.1598 |

NC, not calculated

[1] Difference among the treatment groups (Fisher’s exact test).

# **Figure S1. Time to occurrence of the primary composite MACE by treatment cohort**


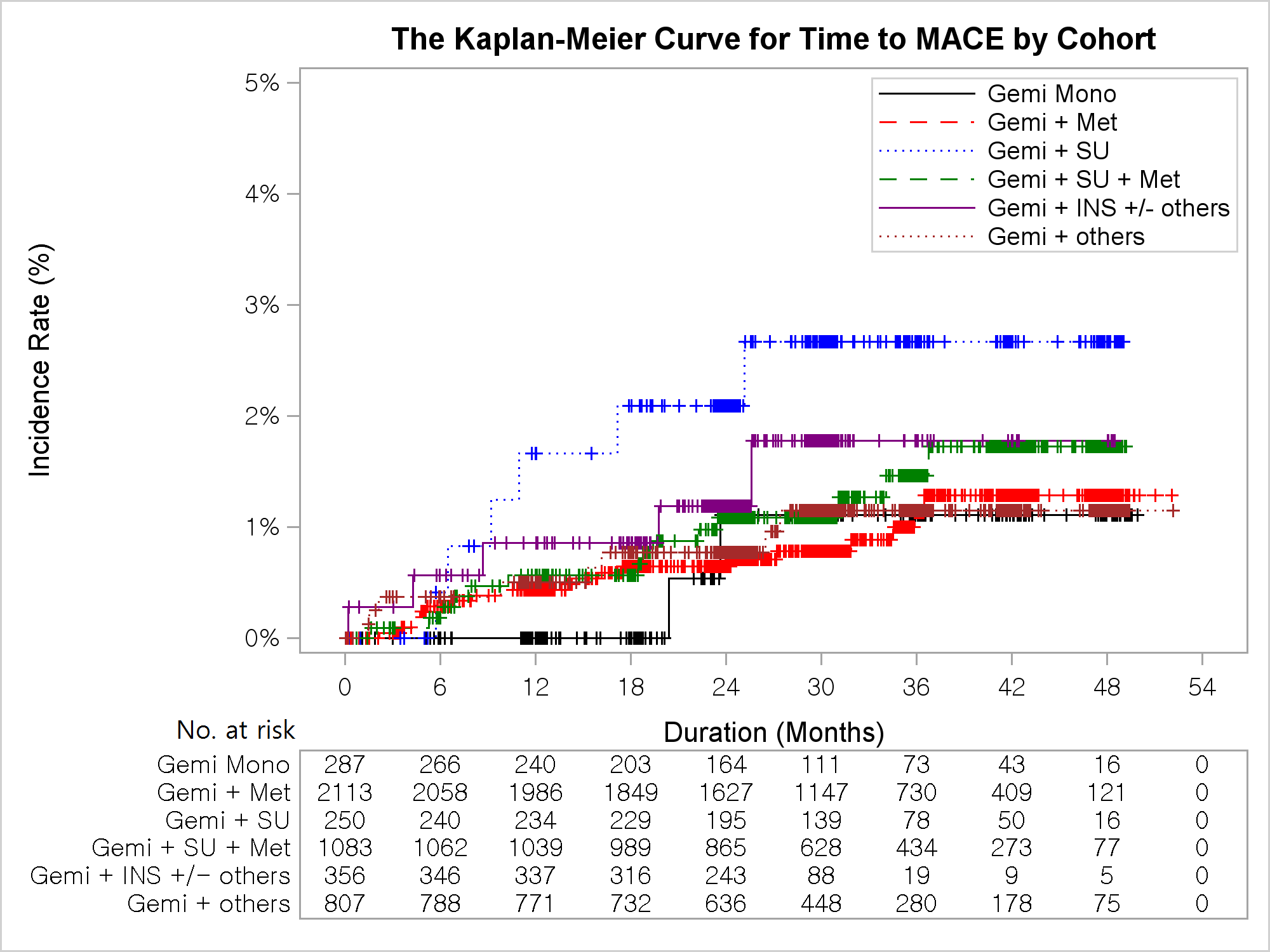


MACE, major adverse cardiovascular event
